# A High-Throughput Small Molecule Screen for *C*. *elegans* Linker Cell Death Inhibitors

**DOI:** 10.1371/journal.pone.0164595

**Published:** 2016-10-07

**Authors:** Andrew R. Schwendeman, Shai Shaham

**Affiliations:** Laboratory of Developmental Genetics, The Rockefeller University, New York, New York, United States of America; East Carolina University, UNITED STATES

## Abstract

Programmed cell death is a ubiquitous process in metazoan development. Apoptosis, one cell death form, has been studied extensively. However, mutations inactivating key mammalian apoptosis regulators do not block most developmental cell culling, suggesting that other cell death pathways are likely important. Recent work in the nematode *Caenorhabditis elegans* identified a non-apoptotic cell death form mediating the demise of the male-specific linker cell. This cell death process (LCD, linker cell-type death) is morphologically conserved, and its molecular effectors also mediate axon degeneration in mammals and *Drosophila*. To develop reagents to manipulate LCD, we established a simple high-throughput screening protocol for interrogating the effects of small molecules on *C*. *elegans* linker cell death *in vivo*. From 23,797 compounds assayed, 11 reproducibly block linker cell death onset. Of these, five induce animal lethality, and six promote a reversible developmental delay. These results provide proof-of principle validation of our screening protocol, demonstrate that developmental progression is required for linker cell death, and suggest that larger scale screens may identify LCD-specific small-molecule regulators that target the LCD execution machinery.

## Introduction

Programmed cell death is an active process that controls cell numbers, eliminates damaged or mutated cells, and contributes to tissue morphogenesis during development. Apoptosis, a well-studied cell death process, is conserved in all animals examined, and is mediated by caspase proteases [1]. Despite its prevalence, apoptosis may not account for a substantial portion of developmental cell elimination. Indeed, mice lacking caspase-3, caspase-9, Apaf-1, or Bax and Bak, key apoptosis regulators, develop to adulthood [2–4]; a puzzling observation given the large number of cells that normally die during murine embryogenesis [5]. Thus, another, caspase-independent, non-apoptotic cell death pathway may also mediate developmental cell death.

The nematode *Caenorhabditis elegans* has proven a useful model for mechanistic studies of cell death. Over the course of *C*. *elegans* development, 131 of 1090 somatic cells generated in the hermaphrodite die, and 147 of 1178 somatic cells generated in the male are eliminated [6,7]. Most of these cell death events are caspase-mediated, and occur within 30 minutes of precursor cell division [8–10]. Furthermore, most dying cells in *C*. *elegans* are undifferentiated.

The male-specific linker cell is unique among cells fated to die as it persists for far longer before cell death onset (~30 hours), and dies as a well-differentiated cell. The linker cell leads the developing gonad as it migrates from its initial position in the midbody, at the second larval (L2) stage, to the posterior cloacal region in L4 animals. At the L4-to-adult transition, the linker cell dies using a genetic program independent of all known apoptosis genes [11]. A genetic network promoting linker cell death has recently been described in which three parallel pathways, a Wnt pathway, a MAPKK pathway, and a developmental timing pathway, converge on the stress-responsive HSF-1 transcription factor, acting in a stress-independent mode, to initiate cell death. HSF-1 is required for expression of ubiquitin proteasome system components that promote cell death through an E3 ubiquitin ligase complex likely composed of CUL-3/Cullin3, RBX1, BTBD-2, and SIAH-1, all conserved proteins [11–13].

Several observations support the notion that the linker cell employs a destruction program conserved from *C*. *elegans* to vertebrates, which we have termed LCD (linker cell-type death). First, cell death with similar ultrastructure, including nuclear crenellation, swelling of endoplasmic reticulum and mitochnodria, and lack of chromatin condensation, is common during embryonic vertebrate development and is characteristic of neuronal degeneration in mouse models and human patients with polyglutamine and other neurodegenerative diseases [14–17]. Second, the self-aggregating *C*. *elegans* glutamine-rich protein PQN-41C is required for LCD, reminiscent of aggregation of abnormal glutamine-rich repeat proteins that promote neurodegeneration in a variety of human diseases (e.g. Huntington’s disease) [12,16,18]. Third, severing of neuronal axons in the mouse leads to cell soma degeneration accompanied by crenellated nuclei [19], as well as distal process degeneration. The latter requires the kinase regulator, Sarm, whose *C*. *elegans* homolog, TIR-1, is a component of the MAPKK pathway promoting LCD [12,20]. Fourth, the MAPKK SEK-1 and its associated protein TIR-1/Sarm are required for human TDP-43/FUS-induced motor neuron degeneration in *C*. *elegans* [21].

To develop tools for manipulating LCD, we sought to identify small molecule inhibitors of this process. Such molecules could be used in *C*. *elegans* to study the LCD pathway, and may also serve as tools for labeling and controlling LCD in other animals. To this end, we developed a screening protocol for identifying compounds that affect *C*. *elegans* linker cell death *in vivo*. This has the advantage of avoiding some false positives that emerge from cell culture assays [22,23], such as molecules that have undesired effects on organism viability and/or development. The ease with which *C*. *elegans* can be cultured, and the low cost of such cultures allows large-scale chemical screens to be performed efficiently [22]. Indeed, some antifungal [24,25] and antibacterial [26,27] compounds have been identified using *C*. *elegans* viability as an assay readout [28]. Inhibitors and activators of signaling pathways have also been identified using *C*. *elegans* by measuring pathway output using genetically encoded fluorescent reporters [29–31].

Here, we describe our protocol for identifying LCD inhibitors, and provide proof-of-principle validation of the approach. From a screen of 23,797 compounds we identified 11 small molecules that block linker cell death progression. 5 of these promote animal lethality, and 6 result in a reversible developmental arrest. These results validate our pipeline, demonstrate that developmental progression is required for linker cell death, and suggest that larger scale screens using our method may identify LCD-specific regulators.

## Materials and Methods

### Strains and Media

*C*. *elegans* strains were cultured using standard methods [32]. The *tra-2(ar221) II; xol-1(y9) nsIs65[mig-24*p::Venus*] X* double mutant was cultured at 15°C [33]. Follow-up experiments were performed using *him-5(e1490) qIs56[lag-2*p::GFP*] V* animals where indicated [11,34,35]. Strains were provided by the *Caenorhabditis* Genetics Center.

### Screening Assay

*tra-2(ar221); xol-1(y9) nsIs65* animals were synchronized by bleaching gravid hermaphrodites and allowing the eggs to hatch in M9 buffer overnight at 20°C. Synchronized L1 larvae were plated on 9-cm nematode growth medium (NGM) plates with OP50 *E*. *coli* and grown at 25°C for 32 hours. Animals were then washed off the NGM plates with S Basal medium, washed twice more in S Basal, and then resuspended in S Basal. 25 μl of worm suspension was dispensed into each well of clear-bottom 384-well plates (Greiner) containing test compounds in 10 μl of S Basal, using a MultiDrop Combi Plate Filler, after which a gas-permeable cover (Excel Scientific) was used to seal the plates. The final concentration of test compounds was 10 μM in 35 μl total volume. For all screens, 0.3% dimethyl sulfoxide (DMSO) was used in negative control wells. For all screens other than the initial pilot screen, 3.78 μM Tyrphostin A9 (Sigma) was used in positive control wells. The final concentration of animals in the screening wells was approximately 5 worms/μl. Animals were incubated in screening plates at 25°C for 12 hours, after which the anesthetic levamisole (Acros Organics) was added to each well, for a final concentration of 1 mM, to immobilize the animals for imaging. The plates were scanned on an ImageXpress Velos Laser Scanning Cytometer (Molecular Devices) using a 488 nm excitation wavelength, and fluorescent linker cells were counted using MetaXpress Software (Molecular Devices).

The pilot screen was conducted using the Library of Pharmacologically Active Compounds (LOPAC) library (Sigma) of 1,280 compounds. The Pan Assay Interference Compounds (PAINS) library, consisting of compounds from various sources, was used to test the robustness of the assay against promiscuous compounds. The primary screen used 23,797 compounds from various libraries, including natural products, off-patent drugs, and active pharmacophores purchased from vendors listed in Table 1.

**Table 1:** Commercial sources of compounds screened.

Vendor	Primary Screen Compounds	PAINS Compounds
Chembridge	1411	13
ChemDiv	1408	364
Chem-X-Infinity	352	0
Enamine	14235	308
Life Chemicals	1408	52
Microsource	320	8
Specs	3872	0
Spectrum Chemical	320	95
Vitas-M Laboratory	633	0
AMRI	0	150
Analyticon	0	1
BioFocus	0	3
Cerep	0	14
GreenPharma	0	7
Prestwick	0	12
Sigma	0	29

### Z-factor calculations

To measure assay quality, positive and negative control data were used to calculate Z-Factors for each screening plate using the equation Z-Factor = 1-3(σ_p_+σ_n_)/(μ_p_-μ_n_), where σ is the standard deviation, μ is the mean, p is the positive control, and n is the negative control [36].

### Hit Analysis

Compounds that showed >40% inhibition in the primary screen were selected for visual inspection. Raw visual data from these wells was viewed in ImageXpress and characterized as either “clean” or “false positive”. False positives were primarily wells in which the compound was insoluble and autofluorescent, or wells in which the animals themselves were autofluorescent. Compounds that passed this test were rescreened by manual counting of fluorescent linker cells. The manual counting procedure was identical to the initial screening procedure until the final stage, in which animals were manually pipetted out of the wells onto agar plates and examined under a fluorescent dissecting microscope (Leica) instead of scanning on the Velos instrument. Compounds that showed higher percentages of linker cells than the negative control in this assay were ordered from commercial sources and tested in various assays.

For experiments on agar plates, 200 μl of compound mixture at the appropriate concentration was added to the surface of solid NGM agar and spread with a cell-spreader. Final compound concentration for these assays was calculated using the volume of agar, assuming free diffusion of compound through the agar. Observation of compounds that were colored indicated that this assumption was reasonable.

### Phenotypic Analysis

To measure compound toxicity, animals were grown to the L4 stage and exposed to compounds as described above. After 12 hours, animals were scored for viability by assessing locomotion and observing pharyngeal pumping. For compounds displaying significant toxicity over the negative control, serial dilution was used to identify the LD_50_. LD_50_ values were calculated using Graphpad Prism software.

To measure developmental delay, animals grown and exposed to compounds as described above were viewed under a compound microscope (Leica) and scored for tail development and linker cell survival. Tails were scored as adult if they had shed the L4 cuticle and showed tail ray and fan development.

## Results

### The screening strain and its linker cell death dynamics

**Fig 1:**
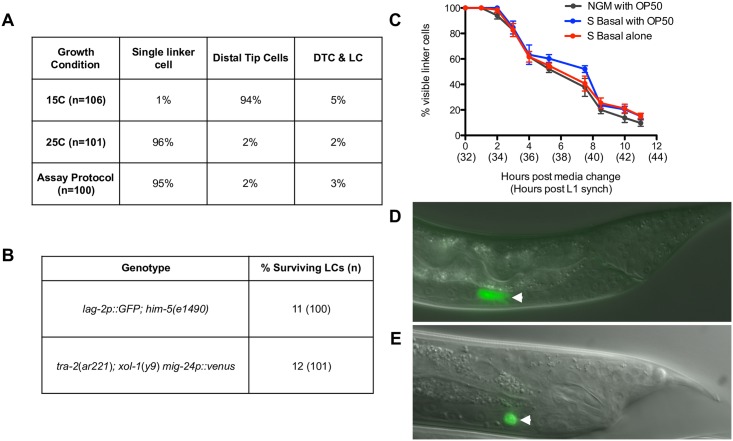
Characteristics of the *tra-2(ar221);xol-1(y9) mig-24*p::Venus screening strain. (A) Percentage of animals with either a single fluorescent linker cell, two fluorescent distal tip cells, or a combination of fluorescent linker and distal tip cells is shown for three growth conditions. For the 15°C and 25°C conditions, gravid adults were placed at the indicated temperature and their progeny scored at the L4 stage. For the assay protocol condition, gravid adults were bleached and their eggs allowed to hatch in M9 overnight at 20°C. Synchronized L1s were then grown at 25°C and scored at the L4 stage. (B) Comparison of linker cell survival in screening strain and standard linker cell death scoring strain. Animals were grown at 25°C and scored for linker cell survival 2–4 hours after the L4-adult transition [12] (C) Percentage of linker cells remaining in *tra-2(ar221);xol-1(y9) mig-24*p::Venus animals over time after an initial growth period of 32 hours on agar plates with food, washing in S Basal, and a change to new growth conditions for 12 additional hours. X-axis numbers indicate hours after media change (no parentheses) and total hours after L1-arrest (parentheses). (D, E) *tra-2(ar221);xol-1(y9) mig-24*p::Venus in migrating (D) and dying (E) linker cells. Arrowheads, linker cell.

To identify small molecules that block linker cell death, we sought to incubate *C*. *elegans* larvae with compounds and identify those that result in a persisting linker cell. We first developed a *C*. *elegans* strain exhibiting a high proportion of males, and in which the linker cell is labeled with a strong fluorescent reporter. Previous studies demonstrated that a *mig-2*4p::Venus fluorescent reporter transgene is specifically expressed in the male linker cell and in the two hermaphrodite distal tip cells [37]. We introduced this transgene into *tra-2(ar221); xol-1(y9)* double mutants, which were reported to develop as XX hermaphrodites at 15°C, and as XX males at 25°C [33]. We found that 94% of animals grown at 15°C possess two fluorescent distal tip cells, suggesting a nearly pure hermaphrodite population; while 96% of animals generated from adult hermaphrodites shifted to 25°C exhibit a single fluorescent linker cell, indicating a nearly pure male population (Fig 1A). Similarly, 95% of embryos grown at 15°C, synchronized at the L1 stage, and then shifted to 25°C appear morphologically male, and have a single fluorescent linker cell (Fig 1A and 1D). Thus, the *tra-2(ar221); xol-1(y9) mig-2*4p::Venus strain is suitable for our screen.

To determine when animals should be incubated with compounds, we tracked linker cell death in synchronized *tra-2(ar221); xol-1(y9) mig-24*p::Venus animals. At 25°C, all synchronized L1 animals, grown on agar plates seeded with OP50 *E*. *coli* for 32 hours, develop to the mid-late L4 stage, and all possess a living linker cell that is migrating towards the cloaca (Fig 1C and 1D). These animals develop to adulthood over the subsequent 12 hours, during which most linker cells die (Fig 1B, 1C and 1E). Some of these dead linker cells are not eliminated by the time of scoring in the standard assay, but can be classified as dead based on morphology, independent of the fact that they express GFP [11]. Development and linker cell death proceed at this stage even when OP50 *E*. *coli* is excluded from the liquid medium (Fig 1C). Using linear regression, we analyzed the rate at which linker cells disappear as the population of animals ages into adulthood, and determined that the rates were not significantly different between agar plates with food and liquid medium with or without food (Slopes -9.2 +/- 0.31, -8.6 +/- 0.38, -8.6 +/- 0.34; p>0.37). These results demonstrate that addition of compounds to *tra-2(ar221); xol-1(y9) mig-24*p::Venus animals at 32 hours post-L1 for 12 hours should allow effects on linker cell death to be revealed.

### Pilot screen design and execution

**Fig 2:**
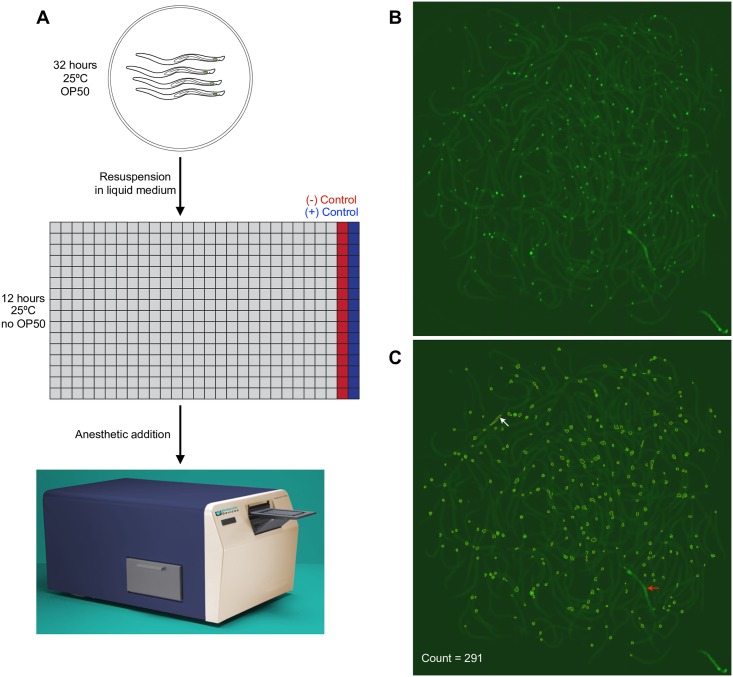
Screening pipeline. (A) Assay workflow: male *tra-2;xol-1 mig-24p*::*Venus* animals grown on agar plates with OP50 at 25°C for 32 hours are resuspended in S-Basal medium and transferred to plates with screening compounds in most wells (grey squares), positive (blue) and negative (red) controls. Plates are incubated for 12 hours and scanned using a fluorescence cytometer. (B) Image of Tyrphostin A9-treated well. (C) Same as (B), except counted objects marked (yellow). Large debris (red arrow) is not counted by the software, though some smaller fluorescent shapes that are not cells are erroneously labeled as cells (white arrow). “Count” indicates object count output from the MetaXpress software.

Guided by these initial studies, we pursued a pilot screen (see Materials and Methods for details). *tra-2(ar221); xol-1(y9) mig-24*p::Venus animals, synchronized at the L1 stage, were grown on NGM plates seeded with OP50 *E*. *coli* for 32 hours at 25°C (Fig 2A). Animals, most of which were phenotypically male, were then washed off the plates and 25 μl of the suspension was added to each well of a 384-well plate containing 10 μl of assay compound. Animals were incubated with the test compounds at 10 μM for 12 hours at 25°C without mixing, and then anesthetized with levamisole to prevent their motion and to allow them to settle to the bottom of the wells. As in other studies [28,30,31,38,39], micromolar compound concentrations were used, because the *C*. *elegans* cuticle acts as a barrier for small molecule access, requiring high concentrations to elicit effects [40,41]. Each plate was then scanned on a cytometer, which counts fluorescent spots, which in our case represented mostly Venus-expressing linker cells (Fig 2B and 2C; S1 Fig).

**Fig 3:**
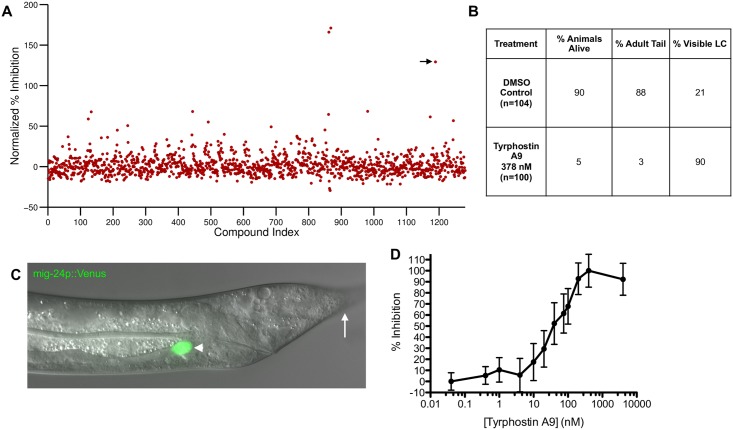
Tyrphostin A9 causes linker cell persistence. (A) Screen of the LOPAC library using Tyrphostin A9 as a positive control. Compounds are plotted with an arbitrary index (X-axis). Percent inhibition (Y-axis) is normalized to negative control and positive control (Tyrphostin A9 treated) counts for each plate screened. The Tyrphostin A9 data point is indicated (arrow). (B) Effects of Tyrphostin A9 on animal viability, development, and linker cell presence are compared to DMSO exposure alone. LC, linker cell. (C) Image of a *tra-2(ar221);xol-1(y9) mig-24*p::Venus animal, treated with Tyrphostin A9 for 12 hours, showing persistent fluorescent linker cell (arrowhead) and undeveloped tail (arrow). (D) Effect on linker cell persistence after 12 hours of treatment with varying concentrations of Tyrphostin A9. Negative control mean is defined as 0% inhibition and maximum linker cell count in Tyrphostin A9 treated animals is defined as 100% inhibition. The EC_50_ for Tyrphostin A9 is calculated to be 45.6 nM.

We screened, in duplicate, the 1,280-compound LOPAC library from Sigma-Aldrich, which includes small molecules with previously characterized activities (Fig 3A, Table 1). Wells with increased cell counts greater than three standard deviations from the negative-control mean in both duplicates were further examined. As shown in Table 2, several of the wells had raw cell counts vastly greater than the average number of animals per well. Scanner images of these wells revealed two causes for this observation. In some cases, the compounds were not fully soluble, and fluorescing precipitate aggregates were erroneously counted by the software as linker cells (S2A Fig). In other wells, animals exhibited high autofluorescence leading to artificially high linker cell counts (S2B Fig).

**Table 2:** Assay positives from pilot screen.

Compound^a^	standard deviations above negative control mean^b^		Well Image Category
	Replicate 1	Replicate 2	
DMSO	0	0	Clean
RU-0024228	12.7	9.7	Autofluorescence
RU-0084525	230.7	80.6	Aggregates
RU-0084544	6.1	6	Autofluorescence
RU-0084546	85.7	116	Autofluorescence
RU-0084567	65.2	44	Aggregates
RU-0084577	4	6.1	Clean
RU-0084629	321.2	159	Aggregates
RU-0084637	24.1	11.3	Autofluorescence
RU-0084675	12.1	8.3	Clean
RU-0084730	17.4	5.3	Clean
Tyrphostin A9	22.6	12.8	Clean

^a^Compounds from the LOPAC library with counts >3 standard deviations above the control mean in two independent experiments.

^b^Values were calculated as (cell count—control mean)/(negative control SD)

### Measuring assay robustness

From the pilot screen, one compound, Tyrphostin A9, resulted in a consistently large increase in the number of fluorescing linker cells, suggesting a possible effect on linker cell death (Table 2). Visual inspection of the Tyrphostin A9 well scans revealed that this effect was not due to issues of solubility or autofluorescence (Fig 2B).

To determine the specificity of Tyrphostin A9 for linker cell death, animals subjected to the screening protocol were recovered from wells prior to cytometry, and examined manually. As shown in Fig 3B, only 5% of animals treated with 10 μM Tyrphostin A9, survived the 12-hour drug incubation period. Animals treated with Tyrphostin A9 also failed to develop adult tail morphology. Nonetheless, linker cells remained strongly fluorescent (Fig 3B and 3C). The effects of Tyrphostin A9 were concentration dependent, with an EC_50_ for linker cell persistence of 45.6 nM (Fig 3D). At all concentrations, linker cell persistence directly correlated with a block in animal development, suggesting that Tyrphostin A9 either inhibits basic cellular processes required for both *C*. *elegans* development and linker cell death, or that blocking *C*. *elegans* developmental progression results in linker cell death inhibition.

Although the effects of Tyrphostin A9 are not specific to the execution phase of linker cell death, we could now use this compound as an effective control to measure assay robustness. We rescreened the LOPAC library (Fig 3A), and used multiple Tyrphostin A9 wells to calculate an average Z-factor of 0.37 (Materials and Methods), indicating a good assay dynamic range [36].

We also screened a small library (PAINS) consisting of compounds arising frequently as non-specific hits across a variety of assays [42,43] (Table 1). Only 1.3% of compounds showed >40% inhibition (S3 Fig), similar to the 1.2% of compounds in the LOPAC screen (Fig 3A).

Taken together, these results demonstrate that our assay design is well poised to identify linker cell death inhibitors, and does not have a propensity to identify false-positives.

### A larger scale screen identifies developmental control compounds affecting linker cell death

**Fig 4:**
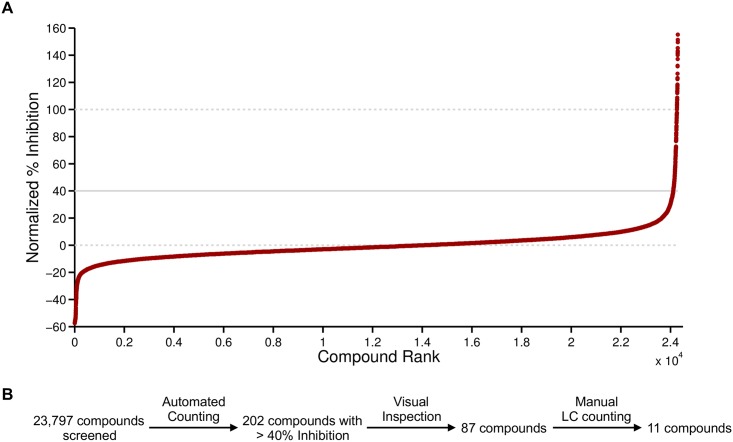
Primary screen results and attrition in secondary analyses. (A) Compounds screened ranked by percent inhibition and normalized to negative and positive control values for each plate screened. All screened compounds are depicted except those with normalized inhibition >160% (23/24298 events). Solid line indicates cutoff for additional testing. Dashed lines indicate percent inhibition of negative controls (0%) and positive controls (100%). (B) Secondary screening resulted in 11 compounds that were further examined.

Using our protocol, we next screened 23,797 compounds from multiple small molecule libraries (Fig 4A; Table 2). Z-factor scores for each plate screened revealed reasonably good dynamic range, with a few exceptions (S4 Fig), with an average Z-factor over all plates of 0.4. 40% inhibition, corresponding to an increase in linker cell survival of more than 3 standard deviations over the negative control mean, was used as a cutoff to select compounds for follow up. 202 of the 23,797 (0.8%) compounds were above this threshold.

Wells for each of the 202 compounds were visually screened for autofluorescent animals, insoluble aggregates, or both (S2 Fig). The remaining 87 compounds were retested manually by scoring linker cell survival under the microscope. 11 compounds reproducibly showed a significant increase in persistent linker cells over the negative control (Table 3).

**Table 3:** Compounds reproducibly resulting in persistent linker cells.

Compound^a^	% Inhibition in Primary Screen	% Visible LC (manual screening assay)^b^	% live LC (2–4 hr adults)^c^	% visible LC (2-4hr adults)^d^	Phenotype
DMSO	0	18	5	20	normal
EN1918	76.4	66	5	20	Toxic
Tyrphostin AG 879	129.3	85	0^e^	11^e^	Toxic
CB0146	65.6	52	2^e^	18^e^	Toxic
EN5065	58.8	53	3	25	Toxic
Leflunomide	45.5	36	4	23	Toxic
EN7212	42.6	52	1	19	Developmental delay
EN9834	60.8	59	0	15	Developmental delay
CB8776	54.4	43	2	26	Developmental delay
EN2416	96.2	45	3	18	Developmental delay
CB0736	48.8	57	6	19	Developmental delay
EN1123	69.2	43	6	24	Developmental delay

^a^Compounds without common names were labeled according to commercial source and catalog number for simplicity.

^b^Value is the percentage of animals scored with a fluorescent linker cell visible under a compound microscope. Visible linker cells may be alive or dead, and animals may be L4 larvae or adults. Manual screening was performed by examining animals after 12 hours of compound treatment at 10 μM and under a dissecting microscope. When scoring the % surviving and % present linker cells in adults, animals were treated on agar plates, picked to new plates at the L4-adult transition, and scored at the compound microscope 2–4 hours afterward. n>71 for all scoring experiments. LC, linker cell.

^c^Value is the percentage of adult animals with a surviving linker cell.

^d^Value is the percentage of adult animals with a fluorescent linker cell visible under a compound microscope. Visible linker cells may be alive or dead.

^e^Tyrphostin AG 879 and CB0146 were treated in the LC assays 250 nM and 2.5 μM, respectively.

**Fig 5:**
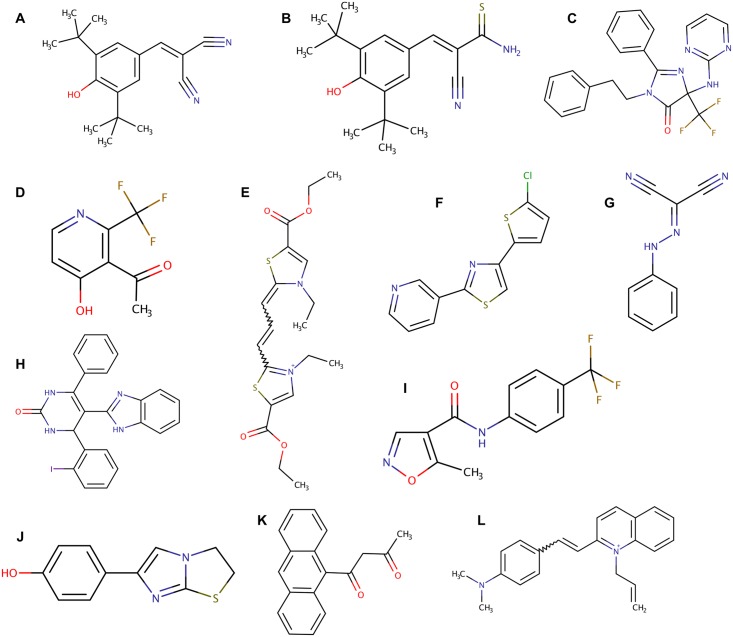
Compound Structures. Chemical structures of compounds identified from pilot screen (A, B) and primary screen (B-M). (A) Tyrphostin A9, (B) Tyrphostin AG 879, (C) CB8776, (D) CB0146, (E) EN9834, (F) EN5065, (G) EN1918, (H) EN7212, (I) Leflunomide, (J) EN2416, (K) CB0736, (L) EN1123.

**Fig 6:**
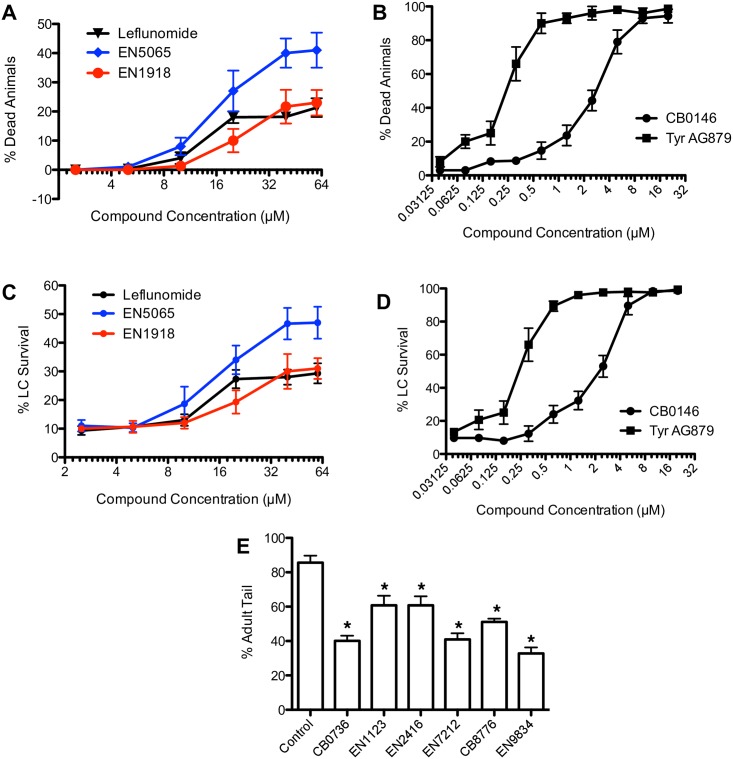
Effects of compound treatment on viability and development. (A) Toxicity following 12-hour incubation with Leflunomide (black), EN5065 (blue), or EN1918 (red) on agar plates with OP50 *E*. *coli*, starting 32 hours after L1 arrest. Animals classified as dead did not move and exhibited no pharyngeal pumping. Error bars, SD. (B) Toxicity of CB0146 (squares) and Tyrphostin AG 879 (circles) as in (A). LD50 values calculated at 2.8 μM for CB0146 and 260 nM for Tyrphostin AG 879. (C) Surviving LCs scored in animals after 12-hour treatment with Leflunomide (black), EN5065 (blue), or EN1918 (red) across a range of compound concentrations. Error bars, SD. (D) Surviving LCs scored in animals after 12-hour treatment with CB0146 (squares) and Tyrphostin AG 879 (circles) across a range of compound concentrations. Error bars, SD. (E) Effect of compounds on male tail development in liquid assay. Error bars, SD. *, p<0.01, student’s t-test.

These 11 compounds (Fig 5) were obtained from commercial sources, and their effects were characterized in greater detail (Table 3). We found that 5/11 compounds inhibited animal locomotion and pharyngeal pumping, and many of these animals arrested as L4 larvae and did not develop to adulthood. The effects of these compounds were not reversible. For three of these five compounds, Leflunomide, EN5065, and EN1918, enough animals survived treatment, allowing us to score adults for linker cell death defects (Fig 6A). None of these compounds elicited linker cell or linker cell corpse persistence in adult animals compared to controls. Thus, it is likely that the effects of these compounds on linker cell death are related to their overall effects on animal toxicity. Consistent with this notion, serial dilution of these compounds reduced toxicity, and this strongly correlated with a reduction in linker cell persistence (Fig 6A and 6C).

The two remaining compounds, Tyrphostin AG 879 and CB0146, were uniformly lethal at 10 μM. We therefore performed serial dilutions to identify the LD_50_ for each (260 nM and 2.8 μM, respectively; Fig 6B), and used these concentrations to evaluate the linker cell death defects in surviving adults. No effects on linker cell death or clearance were observed (Table 3). Reduction of toxicity by serial dilution also strongly correlated with a reduction in linker cell survival (Fig 6B and 6D)

Six of the 11 compounds we obtained from our screen did not affect animal viability, and did not result in inhibition of locomotion or pharyngeal pumping. A closer examination revealed that all six compounds delayed animal development, such that animals did not progress to adulthood during the drug incubations. Thus, while 88% of negative control animals had adult tail morphology following a 12-hour DMSO incubation, only 38–61% of animals treated with these 6 compounds displayed adult tails, a statistically significant effect (Fig 6E). As our previous work demonstrated that developmental cues are required for linker cell death initiation, the developmental delay caused by these drugs likely accounts for the apparent effects on linker cell death. Importantly, the developmental delay caused by all 6 compounds is reversible, as all animals transferred to drug-free agar plates progress to adulthood. These adults then fail to exhibit linker cell survival.

In summary, we identified six compounds that indirectly inhibit linker cell death, likely by blocking a developmental input into the linker cell death program.

## Discussion

LCD is a novel cell death program that may complement apoptosis during animal development. It functions in development in *C*. *elegans* [6,11,44], and at least some of its components are conserved in degenerative processes in mammals [14,15,17,19,20,45–48]. Developing reagents for manipulating LCD is therefore an important goal, as these reagents can help determine how broadly LCD is conserved, under what circumstances it might be functional, and perhaps, down the road, for therapeutic intervention. From a practical standpoint, genetic screens for linker cell death mutants are complicated by the fact that recovered mutant males are likely to be sterile, and thus unable to propagate the genetic lesion of interest. Chemical screening can circumvent this difficulty and allow identification of relevant targets. The studies presented here establish a pipeline for identifying small molecules that inhibit LCD. Our studies provide proof-of-concept for this pipeline, and identify six compounds that appear to block LCD in *C*. *elegans* by interfering with the progression of larval development.

At least three partially redundant pathways (Wnts, MAPKK, and developmental timing pathways) govern linker cell death initiation in *C*. *elegans*. All of these have been implicated in developmental processes. Wnts play key roles in inductive signaling controlling cell fate, in cell migration, and in cellular morphogenesis during development [49–54]; the TIR-1/SARM-SEK-1/MAPKK pathway regulates neuronal differentiation, and appears to be required for early embryonic development [55,56]; and LIN-29/Zn-finger and other components of the heterochronic pathway control developmental timing [57–59]. Since most of the genes controlling linker cell death are components of these pathways, it is, perhaps, not surprising that the non-lethal compounds we identified have broad effects on developmental progression.

Several reasons may underlie our failure to identify linker cell death inhibitors that specifically block LCD execution. First, we may have not screened enough small molecules, in terms of sheer number or diversity. Second, previous studies demonstrate that key execution components of linker cell death are broadly expressed, and their inhibition or activation may therefore lead to toxicity. Third, while small molecules can be excellent inhibitors of enzyme-mediated catalytic reactions, it is possible that linker cell death is governed by a different biochemistry, such as protein-protein interactions, which has been, with key exceptions, difficult to probe with small molecules [60,61]. Fourth, we screened compounds at high concentrations, to allow penetration of the *C*. *elegans* cuticle, and this may have masked compounds that are toxic at high levels, but that exert more specific effects at lower concentrations. Fifth, it is possible that linker cell death is controlled by redundant processes, and therefore blocking any single process, while having some effect, is outside the dynamic range of the assay.

One way to increase the sensitivity of the screen, allowing the use of compounds at lower concentrations, and circumventing redundancy issues, may be to perform the screen in a sensitized background where linker cell death is already somewhat compromised. Indeed, many mutants exist which block linker cell death in only ~20% of animals. Performing a screen in such a background, however, would likely decrease the dynamic range of the assay, and would require further optimization and careful examination of the sources of variability in the assay.

Two compounds we identified, Tyrphostin A9 and Tyrphostin AG879, have well-characterized biological targets, including tyrosine kinases. Whether such proteins are relevant for linker cell death initiation is not known; however, it is intriguing to note that a role for the MAPKK SEK-1 in linker cell death is known, and SEK-1 affects broad developmental programs [12,55,56]. The upstream kinases for SEK-1 are known during neuronal differentiation, and are not tyrosine kinases. However, none of these upstream regulators control linker cell death, leaving the possibility open that this class of kinases is involved.

Finally, although Tyrphostin A9 and the other compounds we identified do not affect LCD specifically, the toxicity and developmental delay effects of these compounds raise the possibility that they could be used as anthelminths. Nematodes account for a number of human ailments, and are costly agricultural pests. Identification of cheap small molecules that can be used to control nematode growth may therefore be of significance. While the toxicity to humans and other mammals of most of the compounds we identified is unknown, Leflunomide is a pyrimidine synthesis inhibitor used in the treatment of Rheumatoid Arthritis and Psoriatic Arthritis [62]. None of the other compounds we identified have known biological targets, making it difficult to speculate on their potential toxicity to other animals, including humans. More work will be required to determine which of these compounds, if any, show promise for use either in treating human disease or as agricultural pesticides.

## Supporting Information

S1 Fig Scanner image of negative control well. Scanner image of a DMSO-treated well showing few remaining linker cells.
(TIF)

S2 Fig Scanner images of false positive wells. In these cases, the cytometer software reported a high cell count from software (Count), however, most of the objects recognized (yellow outlines) are not linker cells. (A) Well in which software recognized fluorescent objects not associated with animals, which are likely compound aggregates. (B) Well in which software counted objects that are not round linker cells, likely a result of animal autofluorescence interfering with the counting system.
(TIF)

S3 Fig Testing assay on the PAINS library. Linker cell death inhibition scores for 1056 compounds in the PAINS library of promiscuous compounds, ranked by percent inhibition normalized to negative and positive controls.
(TIF)

S4 Fig Z-factors of screen plates. Z-factors were calculated for each 384-well plate used in the main screen using positive and negative controls values from columns 23 (negative) and 24 (positive). Dashed line at 0.4 marks the average Z-factor over all 70 tested plates.
(TIF)
