# Supplementary figures and images for: A High-Throughput Small Molecule Screen for C. elegans Linker Cell Death Inhibitors
Source: PLoS One. 2016 Oct 7;11(10):e0164595. doi: 10.1371/journal.pone.0164595 (PMC5055323; doi:10.1371/journal.pone.0164595)

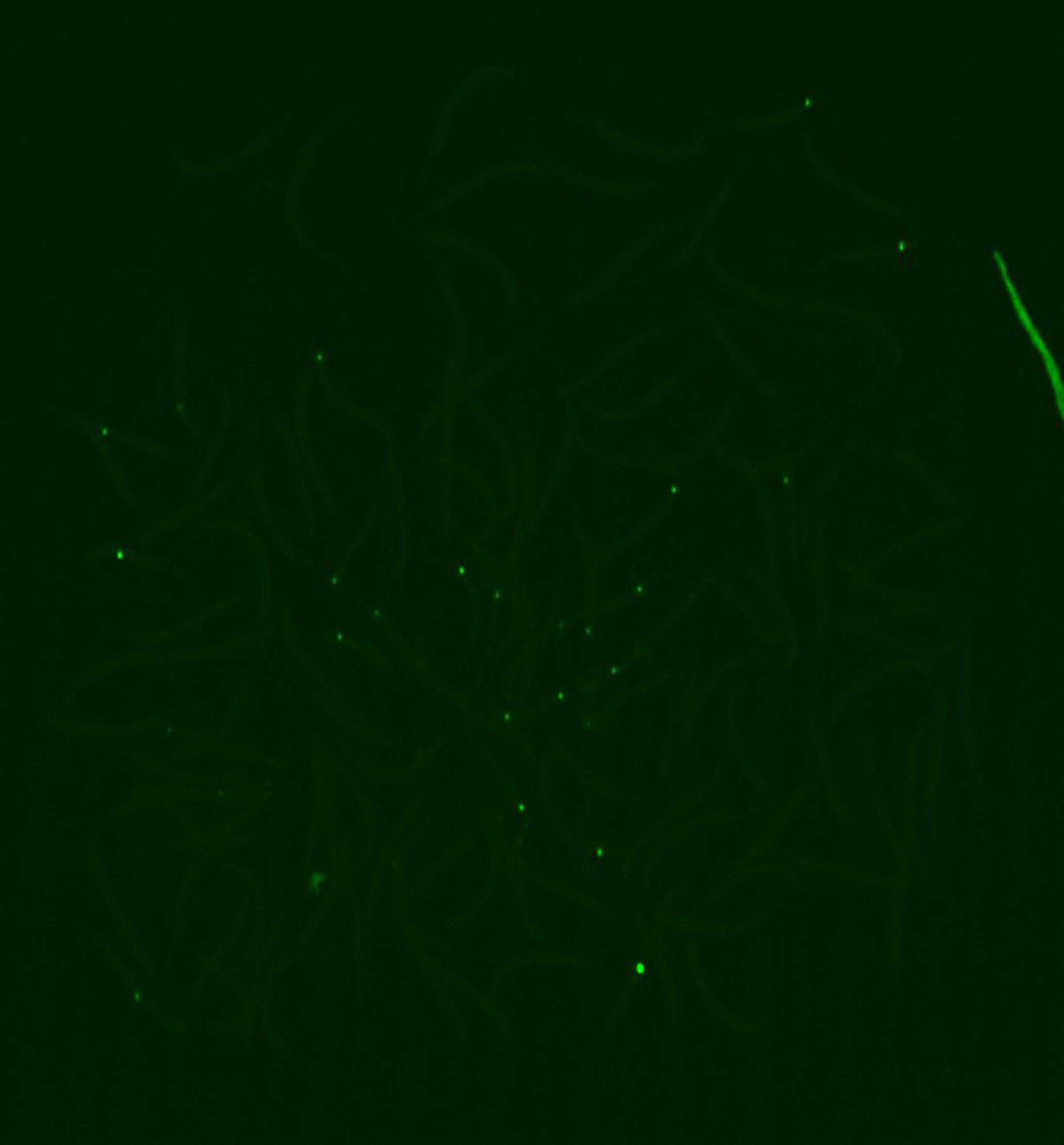

Supplement: S1 Fig — Scanner image of a DMSO-treated well showing few remaining linker cells. (TIF) [file pone.0164595.s001.tif]

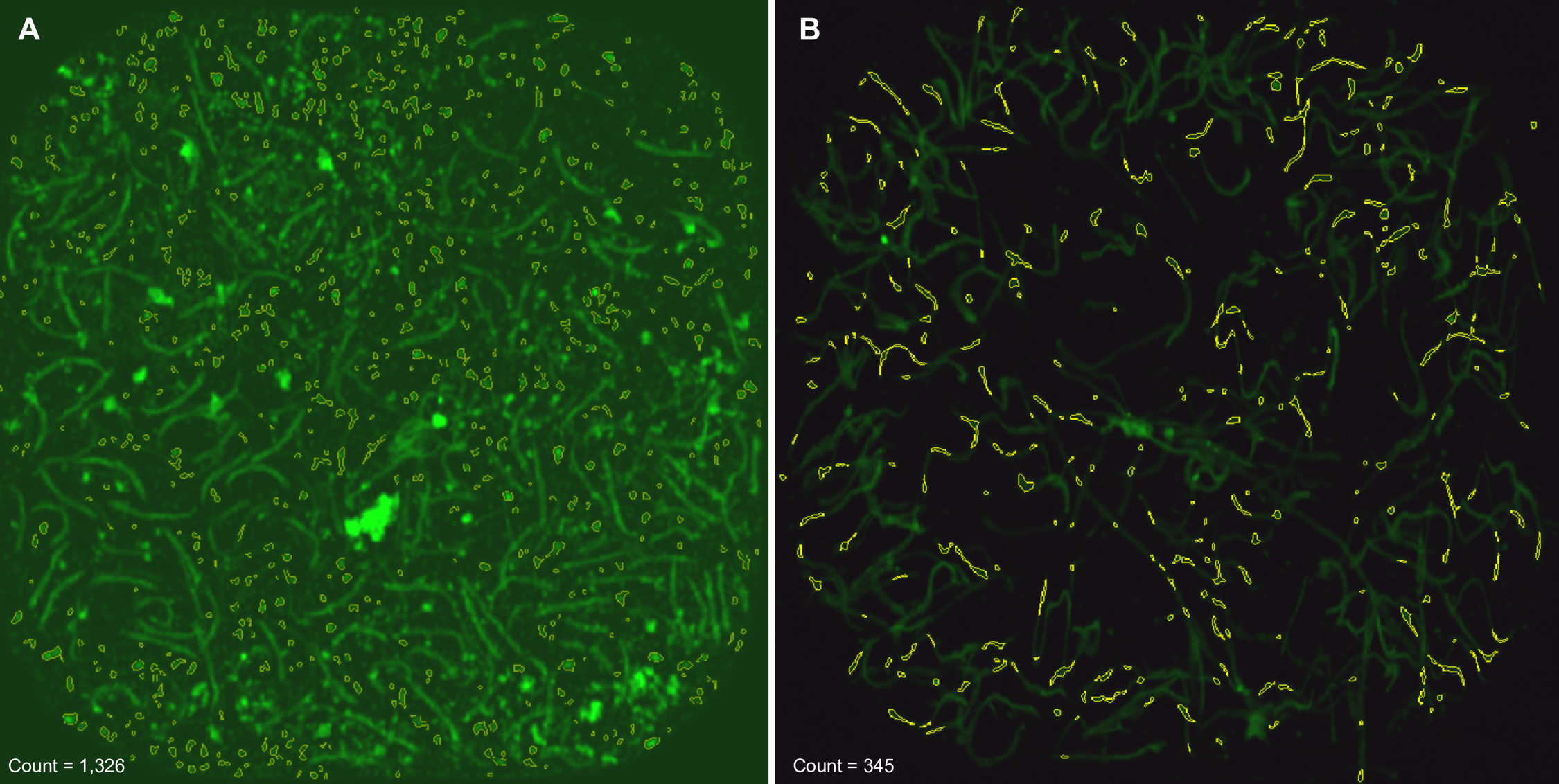

Supplement: S2 Fig — In these cases, the cytometer software reported a high cell count from software (Count), however, most of the objects recognized (yellow outlines) are not linker cells. (A) Well in which software recognized fluorescent objects not associated with animals, which are likely compound aggregates. (B) Well in which software counted objects that are not round linker cells, likely a result of animal autofluorescence interfering with the counting system. (TIF) [file pone.0164595.s002.tif]

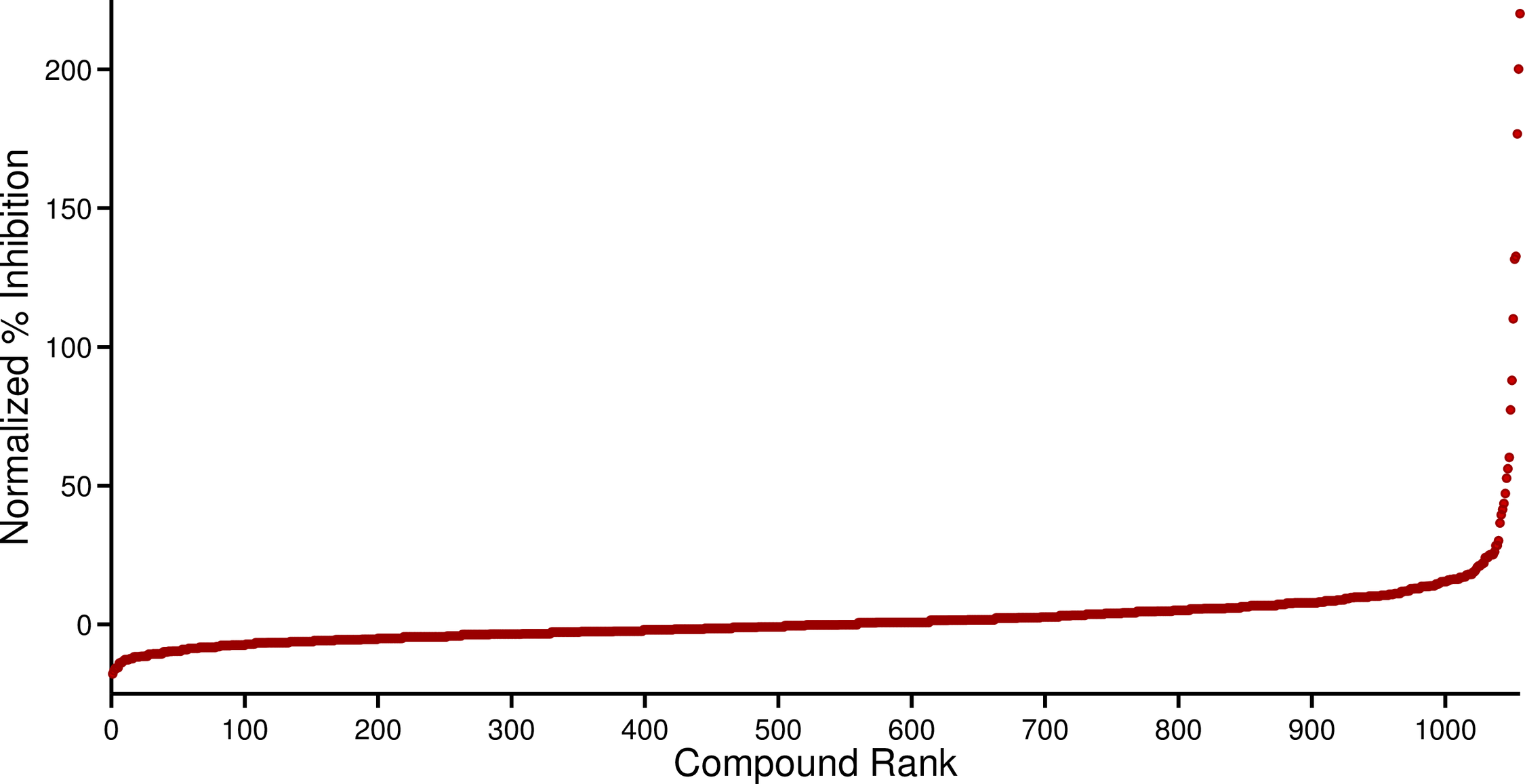

Supplement: S3 Fig — Linker cell death inhibition scores for 1056 compounds in the PAINS library of promiscuous compounds, ranked by percent inhibition normalized to negative and positive controls. (TIF) [file pone.0164595.s003.tif]

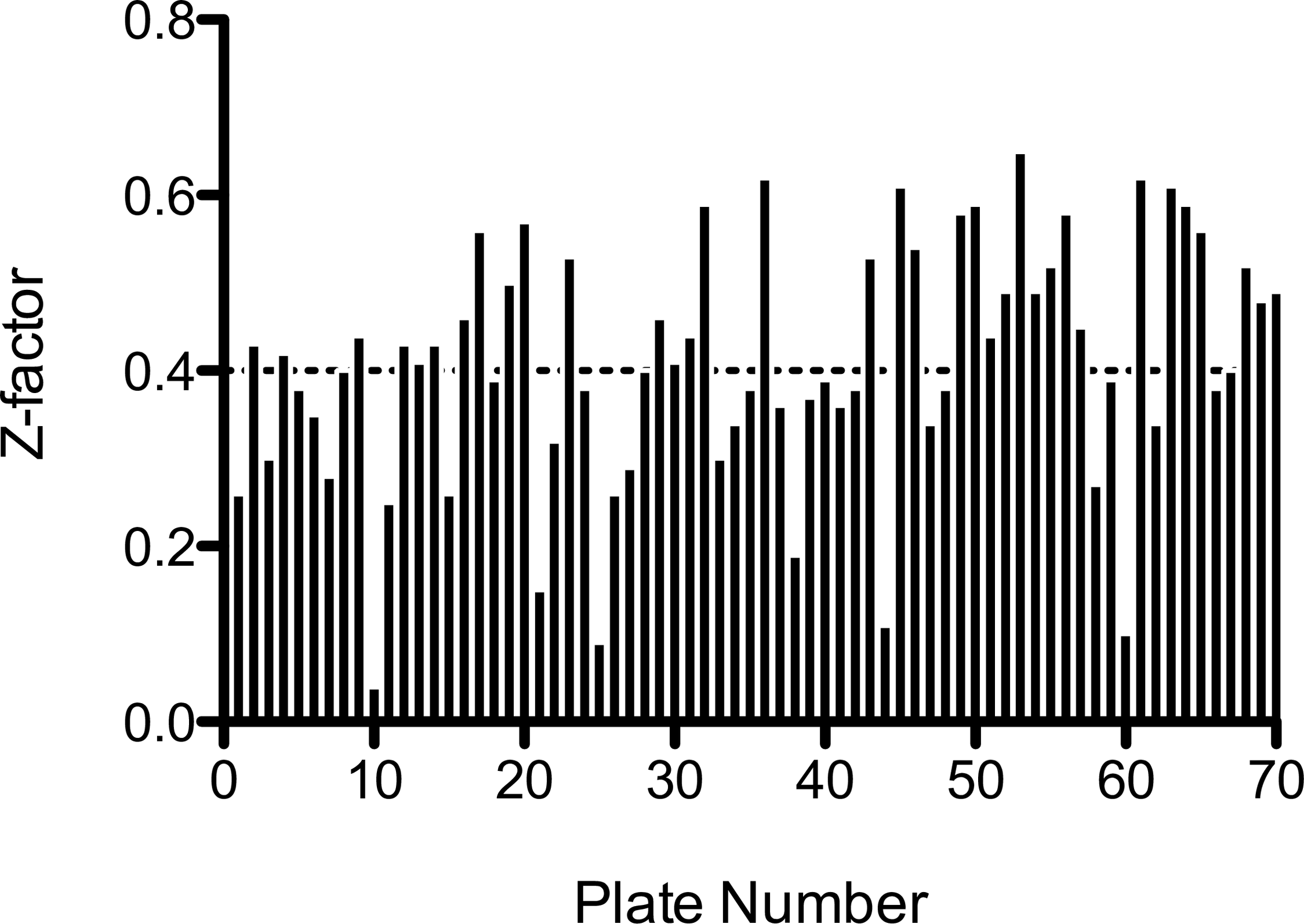

Supplement: S4 Fig — Z-factors were calculated for each 384-well plate used in the main screen using positive and negative controls values from columns 23 (negative) and 24 (positive). Dashed line at 0.4 marks the average Z-factor over all 70 tested plates. (TIF) [file pone.0164595.s004.tif]
